# Community engagement in COVID-19 prevention: experiences from Kilimanjaro region, Northern Tanzania

**DOI:** 10.11604/pamj.supp.2020.35.146.24473

**Published:** 2020-08-14

**Authors:** Innocent Baltazar Mboya, James Samwel Ngocho, Melina Mgongo, Linda Philip Samu, Jeremia Jackson Pyuza, Caroline Amour, Michael Johnson Mahande, Beatrice John Leyaro, Johnston Mukiza George, Rune Nathaniel Philemon, Florida Muro, Jenny Renju, Sia Emmanueli Msuya

**Affiliations:** 1 Department of Epidemiology and Biostatistics, Institute of Public Health, Kilimanjaro Christian Medical University College, P. O. Box 2240, Moshi, Tanzania,; 2 School of Mathematics, Statistics & Computer Science, University of Kwazulu, Natal, Pietermaritzburg, Private Bag X01, Scottsville 3209, South Africa,; 3 Community Health Department, Institute of Public Health, Kilimanjaro Christian Medical University College, P. O. Box 2240, Moshi, Tanzania,; 4 Department of Health, Moshi Municipal Council, P. O. Box 318, Moshi, Tanzania,; 5 Pathology Department, Kilimanjaro Christian Medical Centre, P. O. Box 3010, Moshi, Tanzania,; 6 Mega Afya and Business Company Limited, P. O. Box 6791 Moshi, Tanzania,; 7 Pediatrics Department, Kilimanjaro Christian Medical Centre, P. O. Box 3010, Moshi, Tanzania,; 8 Community Health Department, Kilimanjaro Christian Medical Center, Box 3010, Moshi-Tanzania

**Keywords:** COVID-19, community engagement, community response, public health, experiences, Kilimanjaro, Tanzania

## Abstract

Prevention of exposure to the COVID-19 virus in the general population is an essential strategy to slow community transmission. This paper shares the experiences and challenges of community engagement in COVID-19 prevention in the Kilimanjaro region, Northern Tanzania implemented by our team from the Institute of Public Health (IPH), Kilimanjaro Christian Medical University College (KCMUCo) in collaboration with the COVID-19 response team in the Moshi Municipality. We conducted an education session with the COVID-19 response team and together brainstormed transmission hotspots and which interventions would be most feasible in their settings. The first hotspot identified was crowded local market spaces. Suggested interventions included targeted and mass public health education through the engagement of market opinion leaders, public announcements, and radio shows. We conducted participatory rural appraisal techniques to enable market vendors and clients to visualize two-meter distances and provided a prototype hand-washing facility that was foot operated. We found mass public health educational campaigns essential to inform and update the public about COVID-19 pandemic and to address rumors and misinformation, which hampers compliance with public health interventions. Coordinated efforts among stakeholders in the country are necessary to develop context-specific prevention and case management strategies following the national and international guidelines. Local ownership of recommended interventions is necessary to ensure compliance.

## Commentary

Tanzania had its first reported COVID-19 case on March 16, 2020, a returning national who re-entered the country through the Kilimanjaro International Airport (42kms from Moshi Town) [1]. Tanzania´s initial response to the COVID-19 was isolation and treatment of confirmed and suspected cases, and contact tracing. By mid-April, the Ministry of Health announced that local community transmission COVID-19 was taking place. As of May 28, 2020, Tanzania had 509 confirmed cases and 21 deaths due to COVID-19 [2], although the actual number is likely much higher due to low testing coverage. Public health preventive measures began to be implemented with varying degrees of coverage and compliance. Measures included banning all large public gatherings, limiting the number of people attending burials, closure of schools and colleges, installation of hand-washing facilities in public places and households, physical distancing recommendations, and wearing of face masks in high-density settings like markets and on public transport. Notably, religious services were allowed to continue, and Tanzania, unlike regional neighbors, did not enforce a physical lockdown. In actual fact, people were strongly encouraged to continue with income-generating activities. In this context, efforts were urgently needed to support national efforts and implement practical public health interventions to reduce the risks posed by on-going community transmission.

**Figure 1:**
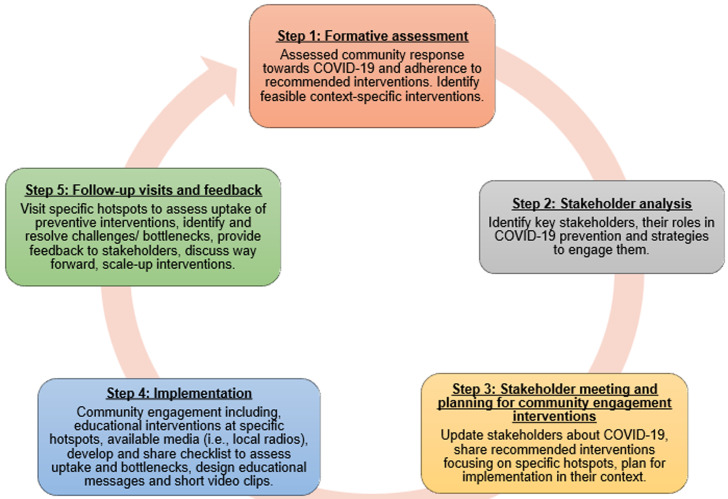
stakeholder engagement steps for public health prevention of COVID-19

**Community engagement activities:** the IPH-KCMUCo team took a 5-steps approach to assess, engage, implement, and sustain public health prevention interventions within the Moshi municipality (Figure 1). During the third week into the Tanzanian epidemic, we conducted formative research to explore community perceptions on transmission, cause, adherence to preventive messages, and perceived hotspots for COVID-19 transmission. These findings guided subsequent actions. Our team then engaged with the Moshi municipal COVID-19 response team under the leadership of the District Medical Officer (DMO) on April 19, 2020. The Moshi municipality is responsible for coordinating all preventive activities at the district, advising the district, and regional leadership through the COVID-19 response team, and have the authority to implement and enforce by-laws and directives to business and other enterprises. During our meeting, we presented the findings from the formative work and further brainstormed critical hotspots for COVID-19 transmission in the municipality. Co-identification of hotspots such as markets, bars and hotels, salons, public transportation, and places of worship, promoted ownership of subsequent activities. Meeting participants identified two key groups of stakeholders to engage, namely the ward health officers and the leaders of all the big markets in the municipality. Ward health officers are responsible for implementing and guiding all health related-preventive activities in the ward. Market leaders have a voice and lead all traders in the respective markets. It also deemed essential to engage the general public through media such as radio, to disseminate appropriate COVID-19 preventive measures and overcoming misconceptions in the community

After the stakeholder engagement, the municipality organized two meetings, one between IPH-KCMUCo, the municipal COVID-19 team, and the 21 ward health officers (at the Moshi Municipality) held on April 22, 2020, and the other with all market leaders (at one of the big markets) held on April 24, 2020. During these meetings, the team conducted an education session, followed by brainstorming with the group to identify which of the recommended interventions [2-5] would be feasible within the different hot spots in their settings. Health officers wanted to start with bars, local clubs, and restaurants, including informal food vendors. The availability of hand-washing facilities, the use of face masks by all workers, physical distancing such as the arrangement of tables giving a space of one meter or more, and disinfection of surfaces before and after customers leave, were identified as feasible in most settings within the municipality. Health officers planned to visit the motorcycles and bus stands, small markets, and saloons to advise accordingly. Planned follow-up visits to restaurants and bars were identified as being important to promote compliance. Similarly, meetings with market leaders started with a short education session of critical aspects of COVID-19, followed by brainstorming of practical interventions at each specific market. The resolution was to ensure the availability of hand-washing facilities at all entries and inside the market, wearing face masks (both sellers/ traders and buyers/ customers), and where possible, the physical distance of one meter between traders and traders with customers. It was also agreed to have a written public announcement, especially the use of face masks and the enforcement of fines for non-compliance. After the meeting at the market, the whole team went around the central market, collecting trader recommendations, and discussing implementation strategies. The team provided a practical illustration of physical distancing, using chalk markings to depict where customers should stand or wait and explained why such an intervention was necessary.

**Figure 2:**
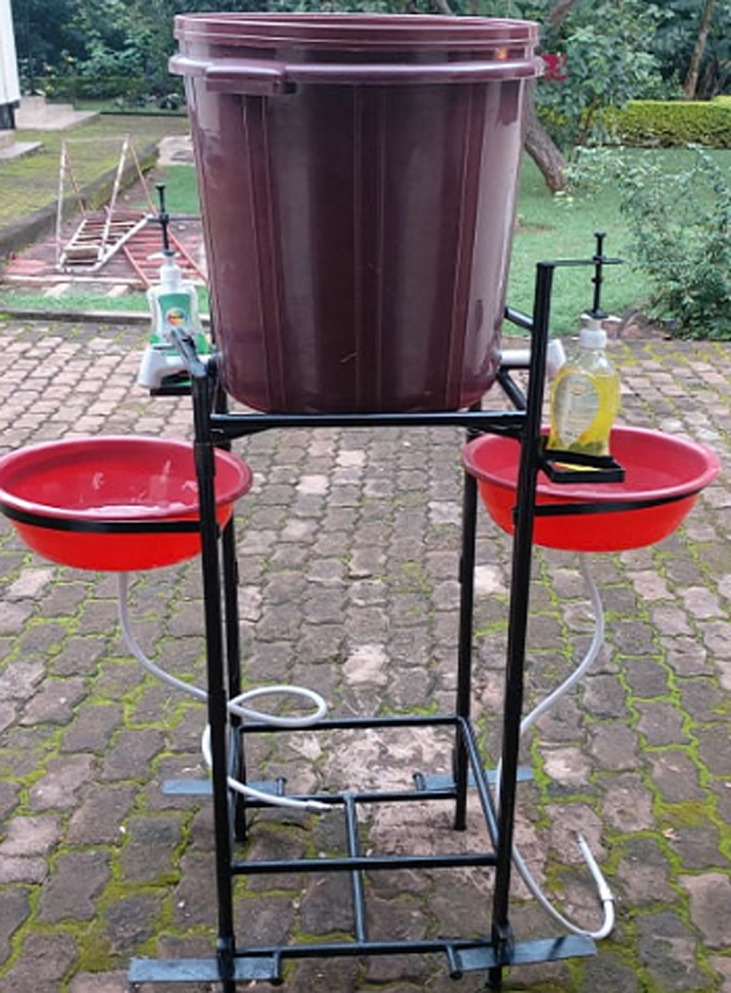
a hand-washing device that uses a foot pump for dispensing both the water and liquid soap donated by IPH-KCMUCo to the central market in Moshi municipality and ward executive office

The IPH-KCMUCo team, together with health officers from the municipality, and ward executive officer, visited the three big markets 2-7 days after the meetings with leaders to assess progress and provide practical advice to address implementation challenges. During these visits, IPH-KCMUCo donated a hand-washing device that uses a foot pump for dispensing both the water and liquid soap, instead of taps requiring users to touch with possibly contaminated hands (Figure 2). The stand was immediately put at one of the gates at the central market. A proto-type hand-washing facility was also left at the ward executive officer´s office to enable adaptation and implementation in other markets and other areas. Follow-up visits at one of the markets found that some traders and opinion leaders reported resistance to the implementation of recommended interventions, specifically the wearing of face masks. In response, the team met with reluctant local leaders and conducted educative and discussion sessions with them to give them reasons why such measures are essential in the prevention of community transmission of COVID-19. Four days after the meeting with the market leaders in the Moshi municipality, the IPH-KCMUCo team revisited the markets to assess the implementation of recommended interventions. It was a humbling experience to observe that people strictly followed a hand-washing strategy at all four gates of the central market (a closed market set in the middle of town). Traders themselves reminded customers to wash their hands before entering the market. This intervention might have been feasible in this particular market only because of the limited entry points allowing leaders and officials more control. The positive attitudes of traders towards hand-washing were instrumental in promoting compliance.

Observations in the largest market in town (not as enclosed as the central market with multiple entries and exit points and a spill out of traders onto the neighboring streets) revealed that despite the size, the arrangement of tables within the space still allowed for physical distancing strategies to be implemented. However, social distancing was a challenge in more crowded market areas. Our team faced challenges in introducing the same public health measures in the third and most crowded market in Moshi. This market has no walls or gates and, therefore, no physical reminders of what is expected of clients or traders. During the follow-up visits, we observed compliance with some interventions. For example, all traders in markets had face masks. However, they were not wearing them at all times. The same problem was witnessed amongst customers. Therefore, the leadership of the markets instigated a fine for non-compliance to wearing face masks equating to approximately US$3 and two bottles of liquid soap. This was applied to both traders selling goods and customers buying without wearing a face mask. Customers who refused to wash their hands were not allowed inside the market. The sustainability of such an intervention is yet to be established. The team, in collaboration with the Moshi municipality, developed a monitoring checklist to assist health officers in assessing the uptake of recommended interventions, progress, and bottlenecks at different hotspots, particularly bars and restaurants, salons, markets, and public transport stands in their areas. Our team also developed preventive messages in Swahili for each hotspot that can be used by any person involved with COVID-19 prevention and control. A copy of the messages was shared with the municipal COVID-19 response team, ward health officers, and market leaders.

Our activities highlighted the need for public awareness campaigns to focus on creating awareness regarding the potential benefits of the preventive approaches, seriousness, susceptibility of COVID-19, and addressing potential barriers to uptake of the interventions [6-8]. As well as conducting education specific to hotspots, our team provided mass education and information through different media platforms. A small group from IPH-KCMUCo established contact with local radio stations (Radio Moshi FM, Kili FM, Radio Voice of Gospel, Radio Kicheko, and Fountain Radio) who provided free consecutive public education sessions, from April 21, 2020, onwards. The willingness of the management of these radios to offer free sessions was due to their readiness to support COVID-19 prevention efforts. These radios also broadcast to neighboring regions, therefore increasing the reach of this mass education. Radio Kicheko also recorded a program that was later used to provide public education. The team also designed and circulated short messages and video clips through social media platforms to educate the public and contribute to correcting the circulating myths. Furthermore, radio programs, which included opportunities for public questions and answers, allowed for essential opportunities to clarify and correct myths surrounding COVID-19 in the community. The need to address false news, and misinformation is also elsewhere emphasized [1,6,7]. According to WHO, misinformation hampers public health responses to epidemics, particularly COVID-19, and prevents people from taking adequate measures to prevent disease transmission [8] effectively.

Religious leaders were also crucial stakeholders in COVID-19 prevention. The IPH-KCMUCo team had meetings with individual religious leaders who are known by members, to discuss potential preventive measures during religious programs. Sessions were held with leaders from different denominations (Catholics, Lutheran, Anglican, Muslims). The recommended interventions, i.e., hand-washing, maintaining a physical distance, disinfection of benches, and surface after services and wearing face masks, have been implemented in the specific places of worship. The collection of these activities contributed significantly to the mass public education and awareness of COVID-19 in the region. Finally, the IPH-KCMUCo team summarized key challenges and provided feedback to the Moshi municipal COVID-19 response team for informed decisions and actions. To this point, there are deliberations about the way forward in this district. The reach of IPH-KCMUCo was further widened through requests from neighboring districts, to provide education to the ward health officers and appear on other local radio stations. The COVID-19 response team in a neighboring district also recommended the formation of a WhatsApp group to include members of the IPH-KCMUCo as well as district officials to be used for sharing experiences, challenges and asking questions. The WhatsApp group facilitated the monitoring of implementation.

**Lesson learned, success, challenges, and way forward: In summary, the key lesson learned included:** 1) Educational interventions are essential to COVID-19 prevention. During the implementation of these activities, the team was able to educate the district through the office of the DMO, motivating uptake of interventions by stakeholders such as health officers and market leaders, seeing the support from local government. 2) Routine follow-up is necessary for hotspots such as markets and places of mass gatherings to enforce uptake of the interventions and to overcome the initial resistance to change. These visits should also include mobilization of opinion leaders and groups of people resisting changes. 3) WhatsApp groups are an effective mechanism to share experiences and challenges among health officers. 4) Support from respective local governments such as the district commissioners, the district/ municipal executive directors, and the DMO is necessary to support and strengthen the implementation of preventive interventions. 5) Question and answer sessions during radio sessions are essential not only for health education but also to allow the public to get context-specific clarifications from health experts. The media stakeholders are willing to participate in disease prevention activities, given that they are timely engaged. 6) The public prefers pictures with brief descriptions or explanations for COVID-19 prevention education over and above short messages alone. There were various successes and challenges in the implementation of community engagement activities in the Kilimanjaro Region. The success included compliance with physical distancing, hand washing, and the wearing of face masks in some settings. The health education through radio shows might have contributed to this success. The radio programs contributed to addressing misinformation and ensuring the public have accurate information and counterarguments against false information [7,9]. The challenges included non-compliance to physical distancing in less organized spaces such as informal markets in the region and a widely witnessed occurrence of incorrect wearing of masks. Wearing face masks is one of the precautionary measures to prevent the spread of the COVID-19 virus [5,10]. Locally appropriate strategies, such as engagement of the market leaders, both official and unofficial, were essential to ensure compliance.

## Conclusion

Mass public health educational campaigns are essential to inform and update the public about COVID-19 pandemic and to address rumors and misinformation, which may hamper the efforts to fight against the virus. Local-level coordination of contextually appropriate strategies is likely to be the cornerstone of the national response. Coordination of regional and district/ council stakeholders such as the regional and district commissioners, local government health management teams, religious leaders, political leaders at all levels, healthcare workers, public health practitioners, the media, and non-governmental organizations, is critical to the effective implementation and ensuring compliance to preventive strategies. Coordinated efforts among stakeholders in the country are necessary to contain COVID-19 in our communities through context-specific prevention strategies. Such interventions should also focus on ensuring compliance with the national and international recommended preventive measures. Until the point of writing this paper, IPH-KCMUCo continued to provide health education to the public through the local radios. However, due to the evolving nature of this pandemic, it is clear that messaging needs to be dynamic and continuously updated in line with growing evidence and changing recommendations [8].

## References

[1] Tarimo CS, Wu J (2020). The first confirmed case of COVID-19 in Tanzania: recommendations based on lesson learned from China. Trop Med Health.

[2] World Health Organization Coronavirus disease 2019 (COVID-19): Situation Report-128.

[3] Adhikari SP, Meng S, Wu YJ, Mao YP, Ye RX, Wang QZ (2020). Epidemiology, causes, clinical manifestation and diagnosis, prevention and control of coronavirus disease (COVID-19) during the early outbreak period: a scoping review. Infect Dis Poverty.

[4] Ebrahim SH, Ahmed QA, Gozzer E, Schlagenhauf P, Memish ZA (2020). COVID-19 and community mitigation strategies in a pandemic. BMJ.

[5] Qian X, Ren R, Wang Y, Guo Y, Fang J, Wu Z-D (2020). Fighting against the common enemy of COVID-19: a practice of building a community with a shared future for mankind. Infect Dis Poverty.

[6] Sarwar F, Panatik SA, Sarwar F (2020). Psychology of Preventive Behavior for COVID-19 outbreak. Journal of Research in Psychology.

[7] Van Bavel JJ, Baicker K, Boggio PS, Capraro V, Cichocka A, Cikara M (2020). Using social and behavioural science to support COVID-19 pandemic response. Nat Hum Behav.

[8] World Health Organization (2020). COVID-19 Strategy Update.

[9] Van den Broucke S (2020). Why health promotion matters to the COVID-19 pandemic, and vice versa. Health Promot Int.

[10] World Health Organization (2020). Considerations in adjusting public health and social measures in the context of COVID-19: Interim guidance.

